# Opioid Prescribing and Utilization Following Isolated Mid-Urethral Sling

**DOI:** 10.7759/cureus.19595

**Published:** 2021-11-15

**Authors:** Alex J Knutson, Brianne M Morgan, Rehan Feroz, Sarah S Boyd, Christy M Stetter, Allen R Kunselman, Jaime B Long

**Affiliations:** 1 Obstetrics and Gynecology, Penn State Health Milton S. Hershey Medical Center, Hershey, USA; 2 Obstetrics and Gynecology, Penn State College of Medicine, Hershey, USA; 3 Medicine, Penn State Health Milton S. Hershey Medical Center, Hershey, USA

**Keywords:** opioid disposal, opioid utilization, postoperative pain control, mid-urethral sling placement, postoperative opioid prescribing

## Abstract

Introduction: Overprescribing by providers is a leading contributor to the opioid crisis. Despite available information regarding the role that physician prescribing plays in the community availability of opioids, guidelines for the management of acute pain remain sparse. This project aims to evaluate opioid prescribing, opioid usage patterns, and postoperative pain control in patients undergoing isolated mid-urethral sling (MUS) placement.

Methods: Patients who underwent isolated MUS placement from March 19, 2019 through March 19, 2020 were contacted by telephone in May 2020 and asked a series of questions examining opioid usage, postoperative pain, what they did with unused opioids, and whether they had received education on disposal techniques. A chart review was utilized to determine the amount of opioid prescribed, the presence of any operative complications, and medical and demographic characteristics of subjects.

Results: A total of 53 subjects met inclusion criteria, of which 31 participated in a phone interview. Of the 53 subjects, 54.7% received a postoperative opioid prescription, and all but two of these subjects filled their prescription. Of the interviewed subjects, only 66.6% who filled a prescription reported using opioids Fifty percent (n=6) of patients that required oxycodone reported use of four tablets (30 morphine milligram equivalents (MMEs)) or less and used for 1-2 days postoperatively. No patient reported using opioids beyond five days. Only 22.2% reported receiving instruction on opioid disposal, and 16.7% returned unused opioids to a disposal center. 87.1% of subjects rated postoperative pain as “better” or “much better” than expected.

Conclusion: Patients undergoing isolated MUS placement require limited amounts of postoperative opioids, if any are needed at all, to achieve satisfactory pain control. Excess prescribed opioids, along with inadequate patient education on proper disposal techniques, may contribute towards opioids that are at risk of diversion for nonmedical use.

## Introduction

Opioid misuse continues to be a crisis in the United States. From the years 2007 through 2015, the number of opioid-related deaths tripled [1]. Overprescribing by providers is a leading contributor to the opioid crisis, with 74% of opioid abusers obtaining their pills either directly from a physician or indirectly through an acquaintance with an opioid prescription [2,3]. This scenario is pervasive amongst patients prescribed opioid for post-operative pain, as more than 70%-80% of prescribed postoperative opioid remains unused and patients are faced with unclear or inconvenient disposal options [4-6]. Despite available information regarding the role that physician prescribing plays in the community availability of opioids, guidelines for the management of acute pain remain sparse [7]. Thus, postoperative opioid prescribing has few standards, wide regional and cultural variations, and inconsistent applications [6,8,9].

Previous studies have analyzed optimal opioid prescribing following surgery across many specialties [10-19]. Within Obstetrics and Gynecology, multiple procedures including hysterectomies, pelvic organ prolapse reconstruction, and cesarean section have been examined to characterize typical post-operative opioid prescribing and usage practices [10-19]. Mid-urethral sling (MUS) placement is the most commonly performed procedure for stress urinary incontinence (SUI) with more than three million placements worldwide since introduction to the market [20-23]. However, there is currently limited data on opioid usage following MUS placement [12,17]. This study examines the use of postoperative opioids vs. the amount prescribed following isolated MUS placement and presents a model for optimization of prescribing practices. The primary objective of this study was to quantify opioid prescribing and use and to evaluate pain control following isolated MUS placement. Secondary objectives are to evaluate education and practice of disposal methods of opioids.

The findings of this research were presented at the Society for Gynecologic Surgeons (SGS) 47th Annual Scientific Meeting in Palm Springs, CA June 27-30, 2021.

## Materials and methods

This retrospective cohort study was approved by the Pennsylvania State University College of Medicine Institutional Review Board (STUDY 00014899). Enterprise Information Management (EIM) was utilized to identify patients who underwent retropubic MUS placement at two Mid-Atlantic tertiary care hospitals within our academic institution’s Female Pelvic Medicine and Reconstructive Surgery Division, consisting of three Urogynecologists, between March 19, 2019 and March 19, 2020. The year prior to the study’s inception was chosen in an effort to limit time from surgery to telephone survey and therefore limit recall bias. Patient charts were queried for the Current Procedural Terminology (CPT) Code corresponding to a sling operation for stress incontinence (57288) and confirmed to have undergone the surgery of interest. Patients were excluded from the study cohort if they underwent any concomitant surgical procedures other than cystoscopy; sustained a significant intraoperative complication which required additional surgery, or had readmission to the hospital for a surgical complication. Patients were surveyed via a telephone interview between May 5, 2020 and May 28, 2020 regarding pain control, opioid use, and opioid disposal techniques immediately following their procedure (Table 1).

**Table 1:** Phone Interview Data Collection Form

Phone Interview
Were you offered a prescription for opioid from your provider? (yes/no)
Did you use opioid medication following surgery? (yes/no)
Amount of opioid used? (type and number of tablets used)
For how many days after surgery did you need to use opioid?
Did you feel that you were given far more (1), more (2), adequate (3), too few (4), or far too few (5) tablets than needed? (scale of 1-5)
Postoperative pain medications used? (acetaminophen, ibuprofen, Naprosyn, other)
Other modalities used for pain control? (heat, ice, pressure)
How would you rate your overall postoperative pain on average? (Rate your pain 0-10, with 0 for no pain and 10 for worst pain imaginable)
How would you rate your overall postoperative pain at its worst? (Rate your pain 0-10, with 0 for no pain and 10 for worst pain imaginable)
Did you feel satisfied with your pain control? (Rate 1-5 for: much worse (1), worse (2), same (3), better (4) or much better (5) than expected)
Did you feel you had the right amount of prescribed opioid to manage your pain? (Rate 1-5 for: much more than needed (1), more than needed (2), right amount (3), less than needed (4), far less than needed (5))
Did you require a trip to an urgent care or office to obtain adequate pain relief? (yes/no)
What did you do with any additional leftover opioid, if prescribed? (kept, discarded in trash, discarded in toilet, returned to opioid disposal center)
Were you instructed on appropriate disposal techniques? (yes/no)

Chart reviews were conducted on all eligible subjects to obtain demographic and pertinent health histories. Demographic information obtained included age, insurance type, veteran status, ethnicity, race, body mass index (BMI), and marital status. Health histories obtained included parity, mental health disorders, chronic pain disorders, history of sexual/physical abuse, diabetes mellitus, prior gynecologic surgeries, and substance history; absence of positive documentation was considered a negative finding. Information pertaining to MUS placement, such as the date of surgery, surgeon, ASA class, estimated blood lost, total operating room (OR) time, resident/fellow involvement, complications, hospital length-of-stay, readmission, voiding trial status, and whether subjects were discharged with a catheter was also obtained. The online Pennsylvania Prescription Drug Monitoring Program (PDMP) was utilized to determine if opioid prescriptions were filled by patients post-procedure. The amount of opioids filled after discharge was converted to oral morphine milligram equivalents (MMEs). Opioids utilized in the hospital prior to discharge were excluded from the analysis.

All subjects were contacted by phone by three researchers. A total of three attempted contacts were made for each subject. A telephone message was not left due to lack of universal voicemail box. For those subjects who were consented to participate in the survey, opioid use was quantified by the total number of opioid tablets used and the number of days that opioids were utilized based on patient knowledge or pill count if opioids remained in the home. Subjects were asked to rate the number of opioid tablets prescribed on a scale of one to five; one representing “far too few” and five representing “far more” opioids. Pain was assessed by an 11-point Likert scale, both on average and at its worst postoperatively. Subjects also rated their overall postoperative pain control on a scale of one to five, with one representing “much worse than expected” and five representing “much better than expected.” Pain management strategies were assessed by asking if subjects used additional acetaminophen, nonsteroidal anti-inflammatory drugs (NSAIDs), heat, ice, pressure, or other forms of pain relief. Lastly, subjects were asked what they did with leftover opioid tablets and if they were instructed on appropriate disposal techniques. We utilized the Research Electronic Data Capture (REDCap, Nashville, TN) application to store all study data [24]. 

Descriptive analyses were performed with continuous data summarized as mean ± standard deviation and categorical data as frequency and percent. Fisher’s exact tests were used to examine the association between binary variables. All analyses were performed using SAS software, version 9.4 (SAS Institute Inc., Cary, NC) with p <0.05 deemed statistically significant. 

## Results

**Figure 1:**
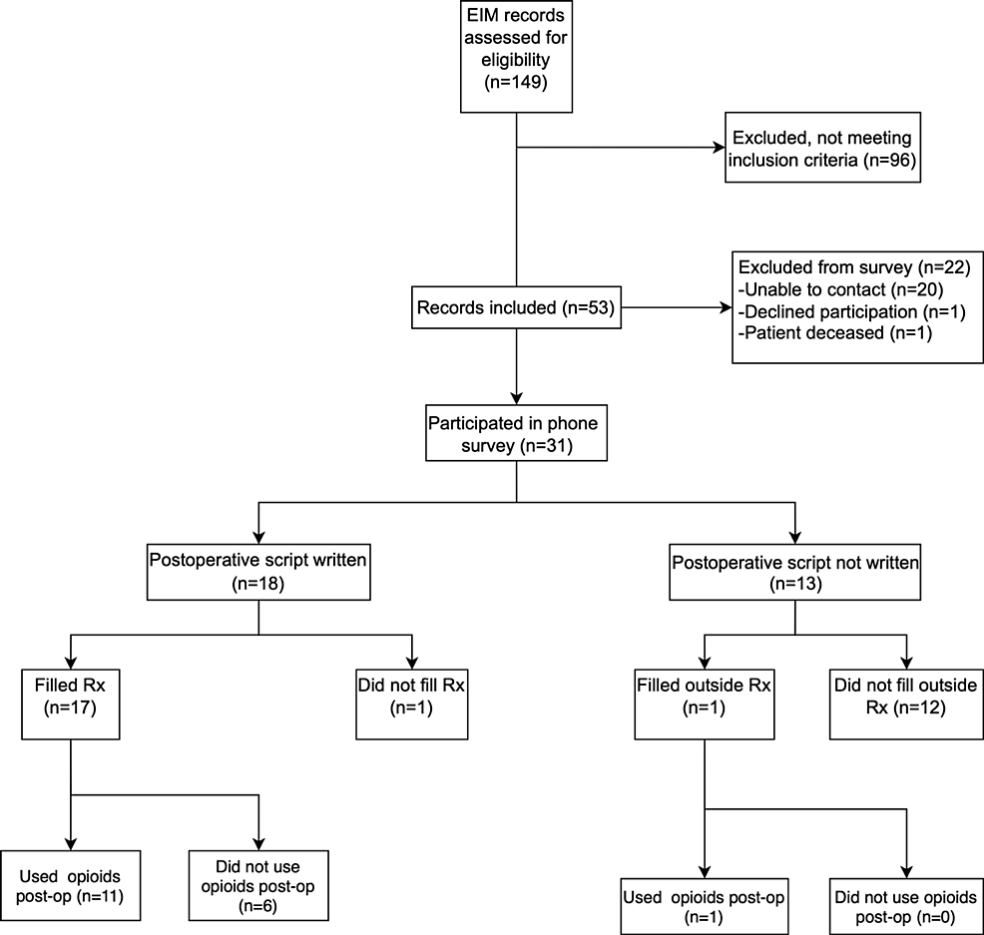
Study Flow Diagram EIM: Enterprise Information Management

EIM records indicated 149 MUS during study dates. A total of 53 subjects met inclusion criteria; subjects that did not meet criteria were due to concomitant procedures. Of these 53 subjects, 31 participated in the telephone survey (Figure 1). Of the 22 patients that did not participate, one declined, one was deceased, and the additional 20 were not able to be contacted. The average time interval from surgery to survey collection was 245 days. The mean age at time of surgery was 52.9 ± 11.6 years; subjects were predominantly white (n=46, 86.8%) and married (n=35, 66.0%). Only one surgical complication was recorded (right dome bladder puncture), and no patients required postoperative inpatient readmission. Four patients failed a postoperative voiding trial and were discharged with an indwelling Foley catheter (Table 2). Demographic characteristics appear to be similar for the 31 survey responders vs. the overall cohort, though rates of diagnosed depression and/or anxiety were slightly lower (41.9% of responders vs. 52.8% of the overall cohort).

**Table 2:** Demographic Data at Time of Surgery mL, milliliters

	All Subjects (n=53)	Interviewed Subjects (n=31)
Age (years)	52.9 ± 11.6	54.0 ± 11.7
Hispanic	4 (7.6%)	2 (6.5%)
Caucasian	46 (86.8%)	29 (93.6%)
Married	35 (66.0%)	23 (74.2%)
Parous	50 (94.3%)	28 (90.3%)
Depression and/or Anxiety	28 (52.8%)	13 (41.9%)
Chronic opioid use	2 (3.9%)	1 (3.2%)
Estimated blood loss (mL)	42.8 ± 26.3	41.5 ± 24.9
Total OR time (min)	24.1 ± 8.5	24.2 ± 7.5
Length of stay (hours)	2.8 ± 1.7	2.7 ± 1.6
Failed voiding trial	4 (7.6%)	3 (9.7%)

Among the 53 total subjects, chart data revealed that 29 (54.7%) were provided a prescription for postoperative opioids from their primary surgeon based upon shared-decision making with patients and surgeons' professional judgment. All patients received standardized pre-operative counseling on pain control. All but two of these subjects filled their prescription according to the Pennsylvania PDMP search. PDMP reliably tracked opioid prescription filling as our cohort was represented entirely by Pennsylvania residents. Additionally, one subject was a long-term opioid user secondary to chronic pain and filled a prescription obtained from her pain management specialist. Across the 53 subjects, the mean MME filled was 39.4 ± 43.4 MME. For reference, five 5-milligram oxycodone tablets is 37.5 MME. The maximum filled was 150 MME (equivalent to twenty 5-milligram oxycodone tablets).

Among the 31 subjects who completed our telephone interview, 18 (58.1%) filled a prescription according to the PDMP search. Notably, only 12 of the 18 subjects (66.7%) who filled a prescription reported taking opioids following their surgery. Of the 12 subjects who reported using opioids, 11 were prescribed oxycodone and one was prescribed hydrocodone. Six subjects reported using four or less 5-mg oxycodone tablets in total, three subjects reported using five, eight and 10 oxycodone tablets, respectively, and the remaining three subjects were unsure of how many opioids they used. Six subjects reported using opioids for only one or two days after surgery; the maximum number of days reported was five. No subjects who used postoperative opioids felt they were prescribed too few opioids; seven (58.3%) felt they were given an adequate amount, while five (41.7%) rated the amount they were given as “more tablets than needed” or “far more tablets than needed.”

On the 11 point Likert pain scale, the mean overall postoperative pain ‘on average’ reported was 3.6 ± 2.4. The mean postoperative pain ‘at its worst’ was 5.5 ± 3.0. Eighteen subjects (58.1%) rated pain much better than expected, nine subjects (29.0%) rated pain better than expected, and four subjects (12.9%) reported pain same/worse/much worse than expected. Use of acetaminophen and/or NSAIDs was reported by 22 subjects (71.0%).

Postoperative pain scores dichotomized as high (>four) versus low (≤four) were associated with opioid use. Four was chosen as the cutoff point due to the average postoperative pain score being 3.6. Eight subjects (66.7%) who used opioids reported high (>four) postoperative pain ‘on average’, compared to three subjects (16.7%) who did not use opioids (p=0.01). Similarly, 11 (91.7%) opioid users reported high (>four) postoperative pain ‘at its worst’, compared to five (27.8%) who did not use opioids (p<.001). Neither postoperative opioid use nor high (>four) pain scores were associated with the use of acetaminophen/NSAIDS. Opioid use was not associated with a medical history of depression and/or anxiety.

Of the subjects who received opioids, eight (44.4%) reported keeping leftover tablets at home, five (27.8%) reported discarding in the toilet or trash, and three (16.7%) returned their excess opioid to a disposal center. One subject used her entire prescription and one subject was uncertain what she did with her leftover opioids. Only four subjects (22.2%) confirmed receiving instruction on disposal techniques.

## Discussion

Our investigation reveals that for most patients undergoing isolated MUS, a limited amount of post-discharge opioid is utilized to achieve satisfactory pain control, and our desire to balance adequate pain control with opioid stewardship more often favors excess opioid dispensation. Among telephone responders, just 12 of 31 subjects reported using opioids following surgery; of these 12, half reported using four 5-milligram oxycodone tablets (30 MME) or less. Postoperative opioids were utilized for five or fewer days after surgery, and half of all users utilized opioids for only two days or less. Despite the low usage of opioids, satisfaction with pain control remained high.

Though there is little existing data on opioid use following isolated MUS procedures, our results are consistent with previous literature examining opioid use both within the broader pelvic reconstructive surgery sphere as well as with isolated midurethral sling. We believe our study represents one of the largest retrospective samples of patients who underwent an isolated MUS procedure for which postoperative opioid prescribing, use and satisfaction was analyzed. A recent study examined, among other procedures, postoperative opioid prescribing and usage among 12 patients who underwent MUS placement, with or without anterior and posterior repair [25]. These subjects were prescribed significantly higher amounts of opioids than they ingested postoperatively. Another study involving a provider educational initiative found a 46.3% reduction in postoperative opioid prescribed following intervention, with no decrease in patient satisfaction between 10 retrospective patients and 21 prospective patients interviewed [17]. Our results add further evidence that isolated MUS patients can achieve satisfactory pain control with no or small quantities of opioid.

Several strategies can help to limit the amount of excess opioid prescribed by encouraging safer prescribing practices through the use of technology. PDMPs allow clinicians to determine the amount of controlled substances obtained by a patient prior to prescribing opioids. Additionally, the use of electronic prescribing modalities can allow clinicians to tailor the number of opioids prescribed to the amount needed. In 2019, the state of Pennsylvania enacted a law requiring that all controlled substances are electronically prescribed. This mandate allows providers to remotely prescribe postoperative opioids after discharge, if needed, thus allowing for more limited amounts of opioid to be prescribed at discharge.

Beyond careful prescribing practices of opioids, clinicians serve a role in reducing the number of excess or unused opioids among their patients through education. Less than 10% of our patients reported disposing of opioids in a safe manner to opioid disposal centers [26-28]. Reasons for this include lack of knowledge regarding proper disposal techniques and a fear of future pain requiring opioid analgesia [29,30]. Among our interviewed subjects who received opioids, only four of 18 (22.2%) affirmed that they were instructed on appropriate disposal techniques, while just three subjects reported returning excess opioid to a disposal center. Future prescribing practices should include educational interventions to increase patient knowledge of proper disposal techniques.

Questions remain regarding how best to implement strategies aimed at balancing postoperative pain needs against the risk of excessive opioid prescribing. Among MUS patients, future studies of educational interventions, including proper disposal techniques, non-opioid pain control methods, and responsible prescribing practices for clinicians, will inform strategies to minimize excess opioids associated with MUS procedures. Prospective evaluation of optimal surgeon prescribing practices as well as specific patient and surgical factors impacting opioid use will continue to promote personalized medicine for our female pelvic medicine and reconstructive surgery (FPMRS) patients.

Our study has limitations that must be considered. First, although our study represents one of the largest published cohort of subjects evaluated for postoperative opioid prescribing and usage following isolated MUS, our sample size is still limited, at 53 total subjects and 31 interviewed subjects. We therefore may lack the statistical power to detect subtle differences in patient characteristics which could illuminate which patients are more or less likely to need opioids in the postoperative period. Second, because our study was a retrospective review with a telephone interview, recall bias could affect the reported numbers of opioids used, how the opioids were disposed of, and overall pain levels. Patients with surgeries that took place a year before data collection are likely at a higher risk of recall bias than patients that underwent surgery following a smaller time interval. We attempted to mitigate this weakness by limiting the cohort to procedures that occurred within one year prior to study inception and utilizing the Pennsylvania PDMP to verify opioid prescription fill and refill. Third, the study was limited by geographical area: subjects had their MUS procedure at one of two hospitals in the same health system located in central Pennsylvania. Thus, our results may not be generalizable to other patient populations in other regions. Finally, there is a potential for non-responder bias. For those patients we could not contact for telephone interview, their overall pain satisfaction or number of prescribed opioids used could be different than those who responded to our telephone inquiries.

In addition to our utilization of the Pennsylvania PDMP to verify opioid prescription and refill rates, additional strengths include the straightforward methodology utilized. By focusing on patients undergoing MUS alone, our study may allow for direct applicability of our findings to practitioners of this patient population.

## Conclusions

Our data supports that patients undergoing isolated MUS placements require limited amounts of postoperative opioids, if any are required at all, to achieve satisfactory postoperative pain control. Excessive postoperative opioid prescribing and inadequate education regarding proper disposal techniques remain opportunities for improvement in our patient care. We recommend that no opioid be prescribed following an isolated MUS unless the patient's needs or preference dictate otherwise. If needed, no more than five 5 mg oxycodone pills should be prescribed. The use of shared decision-making and personalized risk factors for pain may help surgeons balance adequate pain control with the risks of excess opioid prescribing. Future studies are warranted to examine how to best address these issues following MUS placement.
